# Role of the Immune System in the Development of the Central Nervous System

**DOI:** 10.3389/fnins.2019.00916

**Published:** 2019-09-03

**Authors:** Keiko Morimoto, Kazunori Nakajima

**Affiliations:** Department of Anatomy, Keio University School of Medicine, Tokyo, Japan

**Keywords:** MHC, complement, T cell, central nervous system, immune system

## Abstract

The central nervous system (CNS) and the immune system are both intricate and highly organized systems that regulate the entire body, with both sharing certain common features in developmental mechanisms and operational modes. It is known that innate immunity-related molecules, such as cytokines, toll-like receptors, the complement family, and acquired immunity-related molecules, such as the major histocompatibility complex and antibody receptors, are also expressed in the brain and play important roles in brain development. Moreover, although the brain has previously been regarded as an immune-privileged site, it is known to contain lymphatic vessels. Not only microglia but also lymphocytes regulate cognition and play a vital role in the formation of neuronal circuits. This review provides an overview of the function of immune cells and immune molecules in the CNS, with particular emphasis on their effect on neural developmental processes.

## Introduction

**FIGURE 1:**
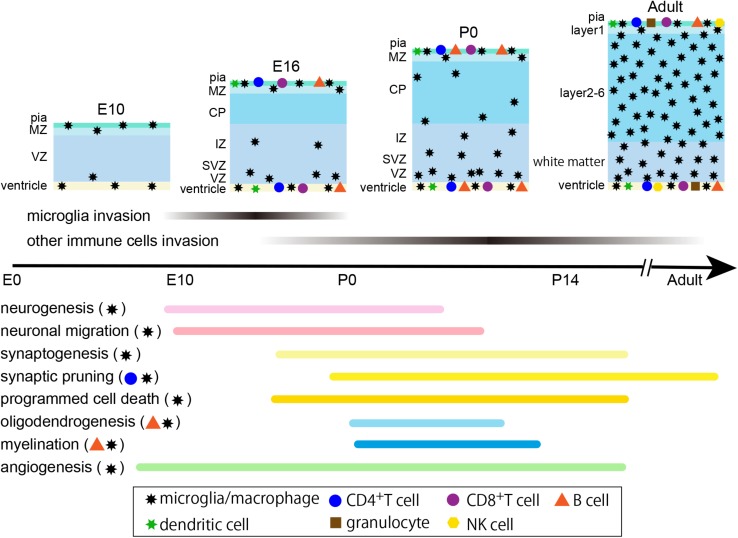
Timeline of cerebral cortex development and distribution of immune cells in mice. Microglia begin to enter the brain at E9.5, and other immune cells, such as T cells, B cells and dendritic cells, infiltrate the brain at least by E16. No data regarding the distribution of granulocytes and NK cells at developmental stages are available; however, they exist in the adult brain. Immune cells, except microglia, are mostly located at the pial surface, ventricle and choroid plexus, and a few cells enter the brain parenchyma. The lower part indicates the time course of major developmental events and the marks on the right illustrate the related immune cells for each process. E, embryonic; P, postnatal; MZ, marginal zone; CP, cortical plate; IZ, intermediate zone; SVZ, subventricular zone; VZ, ventricular zone.

The central nervous system (CNS) and the immune system have much in common. The most prominent characteristic of either system is their ability to transmit information to distant parts of the body with extraordinary specificity and diversity. In the immune system, the diversity of acquired immune cells–T cells and B cells–is generated by the stochastic VDJ recombination of T cell receptors (TCRs) and immunoglobulin (Ig) genes, and somatic hypermutation of TCRs (at least in the shark) (Ott et al., 2018) and immunoglobulins. For example, the human heavy chain region contains 38–46 variable (V) gene segments, 23 diversity (D) gene segments, and 6 joining (J) gene segments, and one segment of each type is selected in each lymphocyte by a mechanism called VDJ recombination. Moreover, the many different combinations of heavy- and light-chain variable regions that pair to form the antigen-binding site result in at least 10^11^ different receptors. The diversity of immunoglobulins is magnified by somatic hypermutation that occurs after the initiation of immune response and introduces point mutations into the rearranged variable region to enhance the reactivity to antigen. As for T cells, theoretically, 10^15^ TCRs can be produced by using almost the same mechanism as for immunoglobulins. This mechanism is critical for the evolution of the vertebrate adaptive immune system, because the genome with its limited size (approximately 3 billion nucleotides) could not directly encode all the possible antigen receptors. On the other hand, the human brain contains approximately 10^11^ neural cells that are classified into hundreds of different neuronal subtypes based on cell morphology, gene expression profile, and axon/dendrite projection patterns. For example, 21 neuronal subtypes are identified in the human frontal cortex by single-cell methylomes (Luo et al., 2017). Each neuron collects inputs from and sends outputs to many other specific neurons–on average, 10^3^ for both inputs and outputs for a mammalian neuron. In addition, glial cells, which outnumber neurons approximately 10 times, cover synapses and control the neural network. It is known that each human astrocyte can contact and encompass nearly 2 × 10^6^ synapses (Oberheim et al., 2006). In this way, specific and diverse neural networks are established, although the precise underlying molecular mechanisms have not been completely illustrated. During the generation of diversity, some non-functional or autoreactive TCR-expressing T cells and undesirable neurons could emerge. These T cells undergo apoptosis in the thymus by a mechanism known as positive and negative selection, and some neurons are removed by apoptosis or lose their synaptic connections through synaptic pruning. Moreover, both systems possess memory mechanisms. In the immune system, after invasion of bacteria, viruses, and other disease-causing organisms, the appropriate acquired immune cells that can respond to specific antigens are expanded and stored as memory T and B cells, so they can immediately generate an accelerated and more robust antibody-mediated immune response when the pathogen is encountered again. On the other hand, synaptic plasticity, including long-term potentiation (LTP) and long-term depression (LTD), underlie memory in the nervous system. In addition, both systems use the mechanisms of accelerators and brakes. In the immune system, there are immunosuppressive T/B cells (Treg, Breg) (Sakaguchi et al., 2008; Rosser and Mauri, 2015) and their imbalance results in allergy and autoimmunity. On the other hand, CNS neurons are classified into excitatory and inhibitory neurons, and the appropriate balance between these two populations is critical for neuronal networks to function normally (Yizhar et al., 2011). During the formation of neural circuits, both excitatory neurons and inhibitory interneurons undergo extensive cell death in the critical window of postnatal development (Southwell et al., 2012) and the survival of interneurons depends on the activity of excitatory pyramidal neurons (Wong et al., 2018). Of note, acquired immune system cells and highly developed myelination in the nervous system appeared at nearly the same time during evolution, around the evolution from jawless to cartilaginous fish (Zalc et al., 2008). It would be interesting if this was not mere coincidence but the two phenomena were causally linked. In addition, our understanding of the CNS has recently dramatically changed from an “immune privileged site” to a “special immune-controlled site.” In 2015, it was discovered that functional lymphatic vessels line the dural sinuses, and are able to carry both fluid and immune cells from the cerebrospinal fluid to the deep cervical lymph nodes (Louveau et al., 2015). The importance of meningeal lymphatic vessels for waste clearance was confirmed because impairment of meningeal lymphatic function caused cognitive impairment in mice (Da Mesquita et al., 2018). These discoveries shed more light on the interaction between the CNS and the immune system. Moreover, emerging evidence suggests that an increasing number of molecules that are typically associated with the immune system are also expressed in various CNS regions and play crucial roles in brain development. This review summarizes the reports on the function of immune cells (Figure 1) and immune molecules (Table 1) mainly in CNS development during the embryonic and early postnatal periods, with some attention paid to their function in more mature brains.

**TABLE 1:** Molecules that are expressed both in the nervous system and immune system, and their reported/potential functions.

**Neuro-immune common molecule**		**Nervous system**			**Immune system**		
		**Expression**	**Function**	**References (bold: review)**	**Expression**	**Function**	**References (bold: review)**
Major histocompatibility complex (MHC) class I	H2-Kb, H2-Db	Neuron, glial cells	Regulate axonal and dendritic growth, synaptic density, synaptic transmission, activity-dependent refinement and plasticity	**Elmer and McAllister, 2012; McAllister, 2014**	All nucleated cells, platelet	Present antigen to T cells, activate NK cells if missing or changed	**Blum et al., 2013; Vivier et al., 2011**
Complement family	C1q, C2-9	Neuron, glial cells	Regulate activity-dependent synaptic pruning, related to neurogenesis, migration, and neuronal survival	**Veerhuis et al., 2011;Stephan et al., 2012**	Epithelial cell, monocyte/macrophage, fibroblast, hepatocyte	Eliminate cellular debris and infectious microbes, orchestrate immune responses	**Ricklin et al., 2010**
	CR3	Microglia			neutrophil, macrophage, NK cell		
Fc receptor	FcγR II B	Purkinje cell	Regulate the development of Purkinje cell	Nakamura et al., 2007	B cell, monocyte/macrophage, neutrophil, dendritic cell, basophil	Low affinity receptor for the Fc region of IgG and negatively regulate receptor-induced signaling	**Bruhns, 2012**
	Fcα/μR	Oligodendrocyte precursor cell (OPC)	Regulate proliferation and maturation of OPC	**Tanabe and Yamashita, 2018a**	B cell, macrophage	Work as a receptor for the Fc region of IgA and IgM	Shibuya et al., 2000
CD3 family	CD3ϵ	Purkinje cell	Regulate the development of Purkinje cell	Nakamura et al., 2007	T cell	Work as a co-receptor for TCR	**Kuhns et al., 2006**
	CD3ζ	dLGN, hippocampal neuron	Regulate activity-dependent synapse formation of RGCs in retina, LTP and LTD, and promote axon pruning	Huh et al., 2000; Baudouin et al., 2008; Xu et al., 2010, **Elmer and McAllister, 2012**			
Cytokine	IL-1β, IL-6, TNF-α, TGF-β	Neuron, microglia, astrocyte	Regulate cell survival, proliferation and differentiation, axonal growth and synaptogenesis	**Bauer et al., 2007; Knuesel et al., 2014**	Several immune cells, fibroblast, endotherial cell	Play key roles in mediating inflammatory and anti-inflammatory reactions	**Arango Duque and Descoteaux, 2014**
Chemokine	CXCL1 (fractalkine)	Neuron	Regulate microglial recruitment, neuronal survival, synaptic maturation, activity and plasticity, synaptic pruning	**Paolicelli et al., 2014**	Monocyte/macrophage, fibroblast, epithelial cell, endothelial cell	Survival, migration and adhesion of monocyte	Imai et al., 1997
	CX3CR1	Microglia			Monocyte/macrophage, T cell subset, platelet, NK cell		
	CXCL12 (SDF-1)	Cerebral cortex (subplate,ventricular surface)	Enhance migration of microglia, NPC, cortical interneuron and Cajal Retzius cell, related to axon guidance, neurite outgrowth	**Li and Ransohoff, 2008; Zhu and Murakami, 2012; Guyon, 2014**	Bone marrow	Essential for development of B cell and homing of hematopoietic stem cell to the bone marrow	**Nagasawa, 2015**
	CXCR4	Neuron			Several immune cells including hematopoietic stem cell		
TLR	TLR2, 3, 4, 8, 9	Neuron, neuronal progenitor cell (NPC), microglia, astrocyte, oligodendrocyte	Related to axon outgrowth, NPC proliferation, cognition, sensory and motor behaviors	**Kioussis and Pachnis, 2009; Okun et al., 2011; Khariv et al., 2013**	Monocyte/macrophage, dendritic cell, B cell, NK cell, regulatory T cell, neutrophil, basophil, fibroblast, epithelial cell, endothelial cell	Key molecules for innate immune system, work as a receptor for peptidoglycan (TLR2), dsRNA (TLR3), LPS (TLR4), ssRNA (TLR8), CpG DNA (TLR9)	**Kawai and Akira, 2007**
Pentraxin	PTX3	Astrocyte	Modulate phagocytic functions of microglia, induce functional synapse formation	Jeon et al., 2010; Fossati et al., 2019	Dendritic cell, macrophage, neutrophil	Activate complement, facilitate pathogen recognition by phagocytes	**Garlanda et al., 2005**
Pcdh	Pcdh18	Ventricular zone in the forbrain and midbrain	Involved in neural circuit formation	**Kim et al., 2011**	Activated CD8+ memory T cell	Function as an inhibitory signaling receptor and restrict the effector phase	Vazquez-Cintron et al., 2012
Dscam	Dscam	Neuron	Specify neuronal wiring, regulate axon guidance and retinal lamination	**Boulanger, 2009; Schmucker and Chen, 2009**	Hemolymph (in flies)	Bind directly to bacteria	Watson et al., 2005
Eph/Ephrin	Ephrin-B1	Neuron	Axon guidance during development, synaptic plasticity	**Klein, 2009**	Germinal center B cell, memory precursor B cell	Inhibit GC recruitment and retention of Tfh cells, promote IL-21 production	Laidlaw et al., 2017; Lu et al., 2017
Semaphorin	Sema3A	Olfactory neuron, cerebral cortex, corpus callosum	Inhibit axon branching in the cortical neurons, regulate pre-target axon sorting of olfactory system	**Tran et al., 2007**; Imai et al., 2009	Dendritic cell, T cell	Inhibit monocyte migration, inihibit T cell activation	**Kumanogoh and Kikutani, 2013; Nishide and Kumanogoh, 2018**

## Contribution of Immune Cells to Cns Function

In the steady state, many lymphocytes reside mostly in the meninges and choroid plexus; however, a few lymphocytes are also found in the brain parenchyma, such as in the fimbria of the dorsal hippocampus and anterior olfactory nucleus, as clearly illustrated by reconstitution of green fluorescent protein-expressing lymphoid cells in *Rag2*^–/–^ mice (Song et al., 2016). The most dominant immune cells in the brain are microglia, which comprise 80% of brain immune cells. Other immune cells identified in the brain include myeloid cells, monocytes/macrophages, dendritic cells, T cells, B cells and natural killer (NK) cells (Korin et al., 2017). Lymphocytes (including T cells, B cells, and NK cells), which are identified as a CD45^hi^ population, are scarce in the CNS, with approximately 10,000 per hemisphere in adult naïve mice (Pösel et al., 2016). However, it is now clear that these limited numbers of immune cells have a significant impact on brain function. In particular, T cells have been implicated in complex brain processes including spatial learning, memory, emotional behavior, and stress responsiveness. For example, in mice undergoing the Morris-water-maze test (MWM), CD4^+^ T cells (helper T cells), but not CD8^+^ T cells (cytotoxic T cells), are recruited to the meninges, and secrete interleukin (IL)-4. IL-4 skews macrophages and microglia to an M2 (anti-inflammatory) phenotype, and induces the production of brain-derived neurotrophic factor by astrocytes, leading to the improvement of spatial learning and memory (Kipnis et al., 2004; Ziv et al., 2006; Wolf et al., 2009; Derecki et al., 2010; Radjavi et al., 2014). Previous studies have also demonstrated that B cells are not required for these processes because B cell-deficient μMT mice do not exhibit learning disabilities (Wolf et al., 2009; Radjavi et al., 2014).

In contrast to the adult brain, data regarding the interaction of immune cells and neural cells— except for microglia—during developmental stages are quite limited. However, epidemiological studies have demonstrated a link between maternal infection and the onset of neurodevelopmental disorders, such as autism spectrum disorder (ASD), schizophrenia, epilepsy, cerebral palsy, anxiety, and major depressive disorder, pointing to the association between the immune system and neural development (reviewed in Knuesel et al., 2014; Estes and McAllister, 2016). Animal models using rodent and non-human primates have also clearly demonstrated a causal relationship between maternal infection and ASD- and schizophrenia-related behavioral abnormalities. It is widely accepted that a major consequence of maternal immune activation (MIA) are changes in microglial morphology, distribution, and the expression level of several marker proteins. Moreover, it is known that microglia have multifaceted functions during normal brain development. It has recently been reported that acquired immune cells are also engaged in the developmental processes of the CNS.

### Neonatal Immune Cell Population

Analyses of embryonic and neonatal immune populations in the CNS remain limited; however, one report illustrated that a small number of lymphocytes infiltrated the developing mouse brain even at embryonic day 16 (E16) and, among the investigated cell types, including CD4^+^ T cells, CD8^+^ T cells, and B cells, B cells are the most abundant in the CNS, peaking at approximately 5% of total lymphocytes (Tanabe and Yamashita, 2018b). Another study, using *CD11c/EYFP* transgenic mice, clearly illustrated that CD11c^+^ (also known as integrin αX and complement component 3 receptor 4 subunit) cells—which include monocytes, macrophages, dendritic cells, granulocytes, and NK cells—were present along the ventricles and within the adjacent parenchyma at E16 and postnatal day 2 (Bulloch et al., 2008). However, there have been no detailed reports describing the subpopulation of immune cells in the developing brain.

### Microglial Function in CNS Development

Microglia are tissue-resident macrophages that play essential roles in innate immunity and have an origin that is different from other immune cells. Hoxb8-negative microglia arise from erythromyeloid precursors in the yolk sack during primitive hematopoiesis and infiltrate the brain at E9.5 in mice (Ginhoux et al., 2010), immediately after the onset of angiogenesis and neurogenesis. In contrast, Hoxb8-positive microglia infiltrate the brain at E12.5 (De et al., 2018). Although microglia are initially located along the meninges and ventricles, after E14 they distribute broadly throughout the cortex and then change their distribution to avoid the cortical plate. After E18, they again enter the cortical plate and begin to distribute to the entire cortex and increase their numbers dramatically (Reemst et al., 2016). During these dynamic changes in microglial distribution, the neural system undertakes highly orchestrated processes, including angiogenesis/vascularization, proliferation and migration of neurons and glia, programed cell death of neural stem cells and neurons, formation of synapses, myelination and establishment of neuronal circuits. Microglia contribute virtually to all of these events (reviewed in Kettenmann et al., 2013; Wu et al., 2015). For example, microglia regulate angiogenesis/vascularization by clearing excess vessels and participating in vessel anastomosis (Fantin et al., 2010), control the number of neural stem cells by phagocytosis (Cunningham et al., 2013), and regulate the survival of neurons in layer 5 via insulin-like growth factor 1 secretion (Ueno et al., 2013). They also modulate major events in forebrain wiring. These include the regulation of invasion of tyrosine hydroxylase-positive dopaminergic interneurons into the subpallium, the laminar positioning of parvalbumin-positive cortical interneurons (Squarzoni et al., 2014), and the control of axon projection through the corpus callosum (Pont-Lezica et al., 2014). Moreover, microglia regulate synaptic formation and synaptic pruning (through activation of the classical complement cascade) (Stevens et al., 2007; Miyamoto et al., 2016); they also regulate myelination by promoting the survival and differentiation of oligodendrocyte precursor cells (OPCs) and the maturation of oligodendrocytes (Pang et al., 2013; Shigemoto-Mogami et al., 2014).

### Role of T Cells During CNS Development

In contrast to the contribution of T cells in adult brain function, very little is known about their function during embryonic and neonatal stages. One of the few explored contributions is their involvement in the pathophysiology of neonatal brain injury. Using postmortem brains from human preterm infants with periventricular leukomalacia, and animal models of preterm brain injury and sepsis-induced white matter brain injury, it was shown that γδ T cells—which have a distinctive TCR and have features of non-MHC-restricted antigen recognition and abundant cytokine secretion capacity—were responsible for injury in the developing brain, and that depletion of γδ T cells resulted in protection from injury (Zhang et al., 2017; Albertsson et al., 2018; Nazmi et al., 2018). Moreover, other groups have also demonstrated that T-helper 17 (Th17) lymphocytes coordinate neuroinflammatory responses in lipopolysaccharide (LPS)-sensitized hypoxic-ischemic injury in neonates (Yang et al., 2014). It has been also reported that *TCR*β ^–/–^γ*^–/–^* mice, which lack both αβ T cells and γδ T cells, exhibit altered size of several areas of the brain and lose sex differences (Rilett et al., 2015). Our knowledge of T cell involvement in CNS development is still fragmented and more work on T cell function in normal CNS development is needed.

### B Cells in Oligodendrogenesis

As mentioned above, B cells accumulate in the neonatal brain and decline in number with age. Most of these B cells are IgM^hi^ B-1a cells (Tanabe and Yamashita, 2018b), which have innate-like characteristics and participate in maintaining tissue homeostasis (Baumgarth, 2011). These B-1a cells are suggested to be mature and are recruited to the meningeal space and lateral ventricle depending on the chemokine receptor CXCR5 and in response to CXCL13 secreted from the choroid plexus. B-1a cells secrete natural IgM antibody and promote the proliferation of OPCs through the Fc receptor for IgM (Fcα/μR). Antibody depletion of B-1a cells diminishes the number of oligodendrocytes and results in reduction of myelinated axons in neonatal mouse brains (Tanabe and Yamashita, 2018b). However, depletion of B-1a cells by antibody treatment also resulted in a decrease in the number of microglia in the subventricular zone. Therefore, it has not yet been completely ruled out that B-1a cells may also affect oligodendrogenesis indirectly through microglia. More detail is provided in Tanabe and Yamashita (2018a, b).

## Molecules That Play Important Roles in Both the Nervous System and the Immune System

It is known that these two systems share molecular mediators of communication in establishing the ability to monitor and respond to changes in the internal milieu and outside environment. Some of these are discussed in this section.

### MHC Class I

MHC class I (MHCI) genes, known to be important for antigen presentation, are polygenic and polymorphic genes, comprising three classes (H2-K, -D, and -L) and multiple variants in mice. They were shown to be expressed in neurons at axons, dendrites and synapses, and in glial cells, especially highly during early postnatal stages. The function of MHCI is well reviewed in Elmer and McAllister (2012) and McAllister (2014). In brief, they are engaged in activity-dependent refinement and plasticity in the visual system, and regulate synaptic plasticity and motor learning in the cerebellum. An important question in this field is whether the diversity of MHCI is necessary for these functions. Synapse elimination and eye-specific axonal segregation in the lateral geniculate nucleus (LGN) were impaired in mice deficient in *H2-K^b^* and *H2-D^b^* (*K^b^D^b–/–^*), and were rescued by expressing a single MHCI molecule H2-D^b^ in neurons (Lee et al., 2014). However, whether each MHCI has specific functions and whether its polymorphism is related to CNS development and cognitive function related to diseases such as autism and schizophrenia remain to be elucidated.

The other major question concerns the MHCI signaling pathway. The TCR is the most widely known receptor for MHCI; however, no TCR protein has been detected in the CNS (Syken and Shatz, 2003). In contrast, CD3ζ, a component of the TCR, is expressed in the LGN during development (described below). Moreover, messenger RNA for PirB (paired Ig-like receptor B), an innate Ig-like transmembrane receptor for MHCI that antagonizes the integrin and MAP kinase signaling cascades (Takai, 2005), is highly expressed in neurons of the cerebral cortex, olfactory bulb, and granule cells of the cerebellum (Syken et al., 2006). Of note, mice deficient for CD3ζ (Huh et al., 2000) or PirB (Syken et al., 2006) also exhibit defects in the activity-dependent refinement of connections, similar to β_2_*m*^–/–^TAP1^–/–^ and *K*^b^*D*^b–^^/^*^–^* mice. Other immune receptors, such as KIR (killer cell immunoglobulin-like receptor) (Bryceson et al., 2005) and Ly49 (a member of the NK family of innate immune receptors) (Zohar et al., 2008), are also believed to be potential neuronal receptors for MHCI. Moreover, whether MHCI molecules in the CNS really present antigens, and if so, what kinds of antigens are presented and whether they are essential for establishing specific neural networks remains to be answered.

### The Complement Family

The classical complement family, an immune pathway that functions to eliminate pathogens and apoptotic cells, also plays an important role in synaptic remodeling. Its function has been reviewed several times, such as in Veerhuis et al. (2011) and Stephan et al. (2012). In brief, complement is produced by neurons and glial cells, especially by microglia and astrocytes, from early developmental periods to adulthood. Complement receptors CR3 (also known as CD11b/CD18, Mac-1, and integrin αMβ2) and CR5 are expressed in resident microglia. These complement proteins are engaged in synaptic refinement of retinal ganglion cell (RGC) projections to the dorsal LGN of the visual thalamus and they are also implicated in neurogenesis, migration, and neuronal survival during development and adulthood. The complement family play these important functions by cooperating with other proteins, because C1q co-localizes with H2-D and H2-K at synapses (Datwani et al., 2009), and the antibody and pentraxin families are involved in the complement cascade, as discussed below. C1q homologous proteins, including C1ql2 and Cbln1, are also engaged in synaptic formation, as reviewed in Südhof (2017) and Yuzaki (2017). However, much remains to be resolved: What signals control the activation of the complement cascade? Are they used similarly during development and adulthood? What are the critical molecules for synaptic pruning that might be the potential pharmacological targets of developmental and neurodegenerative diseases and injuries?

### Antibody Receptors

The blood-brain barrier prevents large molecules, such as antibodies, from entering the brain parenchyma. However, FcγRIIB, a low-affinity membrane receptor for IgG, which negatively regulates B cell receptor-induced signaling, is expressed in Purkinje cells and Bergmann glia in the developing cerebellum. In addition to FcγR, Fcα/μR, a receptor for the Fc region of IgA and IgM, is expressed in OPCs (Nakahara et al., 2003a, b; Tanabe and Yamashita, 2018b). Although the function of these Fc receptors is not yet fully understood, IgG was reportedly detected in the developing rat cortex, and immunohistochemical signals were observed in subplate and other early-generated cortical neurons as well as in retinal and cerebellar neurons during early developmental stages (Upender et al., 1997). The origin of these IgG proteins remains unclear. Because IgG is actively transferred from the mother to the fetus across the placenta using neonatal Fc receptors, and the barrier function of the blood-brain barrier is not complete until E15, maternal antibodies may leak into the fetal brain through blood vessels.

### CD3

CD3 is the most commonly used T cell marker and is composed of four subunits: CD3δ, CD3ε, CD3γ, and CD3ζ. They assemble to form three types of dimers (δε, γε, ζζ), and serve as a co-receptor for MHC-TCR signaling (Call and Wucherpfennig, 2007). CD3ζ is expressed by retinal neurons located in the RGC layer in the developing retina and is localized at synapses in the inner plexiform layer during the period of synapse formation. CD3ζ participates in the eye-specific segregation of RGC axon projections to the dorsal LGN (Huh et al., 2000) and glutamate receptor (GluR)-mediated synaptic activity-dependent synapse formation of RGCs in the retina and dorsal LGN (Xu et al., 2010). CD3ζ is also expressed by hippocampal neurons and the deficiency of CD3ζ results in enhanced LTP and lack of LTD. CD3ζ activation on hippocampal neurons affects cell morphology by promoting dendritic pruning (Huh et al., 2000; Barco et al., 2005; Baudouin et al., 2008). CD3ε is expressed with FcγR II B on Purkinje cells and Bergmann glia in the cerebellar cortex during development, and both CD3ε-deficient mice and FcγR II B-deficient mice exhibit impaired development of Purkinje cells, enhanced paired-pulse facilitation of parallel fiber-Purkinje cell synapses, and poor rotarod performance at high speed (Nakamura et al., 2007). The precise function of these molecules is summarized in a table in Elmer and McAllister (2012). However, the neuron-specific signaling cascade through CD3 and FcγR II B is yet to be uncovered.

### Cytokines

Cytokines are small signaling proteins secreted mostly by immune cells that regulate diverse immunological responses. However, many, such as IL-1α, IL-1β, IL-4, IL-6, IL-10, IL-11, IL-13, IL-18, IL-33, TNF-α, TGF-β and IFN-γ are also expressed in the CNS and are involved in cell survival, proliferation and differentiation, axonal growth and synaptogenesis, as reviewed in Bauer et al. (2007). For example, maternal IL-6 is the central molecule that alters social and cognitive behaviors of the offspring of immune-activated mothers (Smith et al., 2007; Wu et al., 2017), and working memory performance of 2-year-old children can be predicted by measuring maternal IL-6 (Rudolph et al., 2018). It is important to take into consideration that both mother and fetus can produce cytokines, and maternal peripheral and placental cytokines can also reach the fetal brain to directly affect CNS development. If pregnant mice colonized with commensal segmented filamentous bacteria undergo immune activation by infection, high amounts of IL-17a is produced by the mother’s intestinal Th17 cells, is transferred to the fetal brain and binds to IL-17R expressed on neurons, resulting in behavioral and cortical abnormalities that resemble those observed in autism (Choi et al., 2016; Kim et al., 2017; Shin Yim et al., 2017). Moreover, astrocyte-derived IL-33, which is one of the alarmins released by tissue damage, is used for promoting synapse refinement during development (Vainchtein et al., 2018).

### Chemokines

Chemokines are chemotactic cytokines, which direct cell migration, and were originally identified as potent attractants for leukocytes to mediate acute and chronic inflammation. However, accumulating evidence suggests that they also play an essential role in mediating neuron-microglia crosstalk in the developing and mature brains, as illustrated in Ransohoff (2009) and Williams et al. (2014).

One of the most recognized examples is the CX3CL1 (also known as fractalkine) signaling pathway (Paolicelli et al., 2014). Briefly, neuron-derived CX3CL1 and its receptor CX3CR1, which is expressed mostly on microglia, promote microglial recruitment to neuronal circuits by increasing their process movement and cellular migration. This signaling also influences the survival of developing neurons, pruning of synapses, synaptic transmission, synaptic plasticity and connectivity, affecting learning, memory, and behaviors. CXCL12 (also known as stromal cell-derived factor-1, SDF-1)-CXCR4 signaling is also required for the appropriate migration of microglia, neural progenitor cells (NPCs), cortical interneurons, and Cajal Retzius cells. It also controls neurogenesis, axon guidance/pathfinding, neurite outgrowth and maintenance of NPCs, as reviewed in Li and Ransohoff (2008), Zhu and Murakami (2012), and Guyon (2014).

### Toll-Like Receptors

Toll-like receptors (TLRs) are pattern recognition receptors involved in the induction of the innate immune response. There are 13 TLRs identified in mice. Among them, TLR 2, 3, 4, 8, and 9 are expressed in the CNS, and their contribution to various phenomena is suggested, including neurite outgrowth, NPC proliferation, structural plasticity, cognition, anxiety, sensory, and motor behaviors, as discussed in Kioussis and Pachnis (2009), Okun et al. (2011), and Khariv et al. (2013). For example, TLR3 is highly expressed during early developmental stages, its activation reduces embryonic NPC proliferation in the subventricular zone and adult NPC proliferation in the dentate gyrus, and it inhibits neural outgrowth. TLR3 signaling has great impact on cognition; TLR3 deficiency causes improved spatial working memory and contextual fear memory, impaired amygdala-dependent cued fear memory and anxiety. However, the endogenous ligands that activate TLRs under physiological conditions, and whether they affect the development of neural circuits and/or cause more acute effects on synaptic plasticity remain unknown.

### Pentraxins

The pentraxins (PTX) are an evolutionarily conserved family of proteins characterized by a pentraxin protein domain. Some of them, such as C-reactive protein (CRP) and PTX3, are involved in acute immunological responses. It is well known that CRP is a binding partner of C1q and may be involved in synaptic pruning through C1q, and PTX3 can modulate phagocytic activity of microglia and promote functional synapse formation (Jeon et al., 2010; Fossati et al., 2019). Moreover, neural pentraxin 1 (NPTX1) and 2 (NPTX2) mediate synaptic refinement in the developing visual system (Bjartmar et al., 2006) and NPTX2 and neuronal pentraxin receptor (NPTXR) are required for GluA4 expression within parvalbumin-positive fast-spiking interneurons. In *Nptx2^–/–^Nptxr^–/–^* mice, GluA4 is markedly reduced, with consequent reductions in AMPA receptor function in the parvalbumin-positive interneurons, which compromise circuit recruitment of these interneurons, leading to deficits in network rhythmogenesis and behavior (Pelkey et al., 2015).

## Function of Major Neuronal Molecules in the Immune System

In contrast to the molecules that were originally discovered in the immune system and later found to have functions in the nervous system, several molecules, such as Protocadherin (Pcdh), and the Eph/Ephrin and Semaphorin families, were first reported in the nervous system. However, they are also regarded as immune-modulatory molecules. Pcdh18 is an activation marker of CD8^+^ memory T cells that can function as an inhibitory signaling receptor and restrict the effector phase (Vazquez-Cintron et al., 2012). Ephrin-B1(Efnb1) is a specific marker for germinal center (GC) and memory precursor B cells (Laidlaw et al., 2017), and Efnb1 repulsively inhibits GC recruitment and retention of follicular T helper (Tfh) cells. This repulsion requires forward signaling through Eph-B6 on Tfh. Efnb1 also promotes GC Tfh production of IL-21 through forward signaling via Eph-B4 (Lu et al., 2017). Semaphorins, major axon guidance molecules, are also involved in the various phases of physiological and pathological immune responses associated with rheumatoid arthritis, systemic lupus erythematosus, systemic sclerosis and anti-neutrophil cytoplasmic antibody (ANCA)-associated vasculitis. For example, Sema3A synthesized by activated dendritic cells and T cells downregulates T cell proliferation, activates macrophages, and is also involved in DC transmigration across the lymphatics. Sema4A is related to T cell priming and Th1/Th2 regulation and maintenance of Treg stability (Kumanogoh and Kikutani, 2013; Nishide and Kumanogoh, 2018).

## Perspective on Future Directions

As discussed above, the CNS and immune system share many common characteristics; however, some clear fundamental differences do exist, especially with regard to the manner of target molecule/cell recognition. Immune cells can dynamically move around to search for targets and can clonally expand in cell number. The primary means of communication between immune cells include direct contacts with nearby cells that are attracted by chemokines or via secretory molecules. In the nervous system, mature neurons themselves do not proliferate or actively migrate; therefore, where a neuron is located during development is critically important. Moreover, the specific order of signaling between cells in a neural network is essential. For example, when an excitatory neuron A directly projects to neuron B, neuron A would activate neuron B. In contrast, if neuron A indirectly communicates with neuron B through an inhibitory neuron C (i.e., A→C→B), neuron B would be suppressed by the activation of neuron A. The formation of these specific neuronal connections could, if not exclusively, be accomplished by specific and diverse cell adhesion molecules, such as Dscam (Schmucker and Chen, 2009) and clustered Pcdh (Yagi, 2008). For example, the Drosophila *Dscam* gene could theoretically generate 38,016 isoforms (19,008 for the extracellular domain) by alternative splicing. Interestingly, the Dscam protein is also detected in Drosophila immune-competent cells (hemolymph) and is believed to be involved in bacterial binding followed by phagocytosis, suggesting that this molecular diversity may provide a highly diverse innate immune system in insects (Watson et al., 2005). In these examples, extraordinary diversity and specificity, shared by the nervous system and immune system, may be established based on a common molecular machinery or operational modes between these two systems. From this perspective, to understand the mechanisms of development of the complex brain network, significantly more effort should be directed at uncovering why some molecules or cells that are known to work in acquired immunity are expressed/exist in the developing nervous system.

## AUTHOR CONTRIBUTIONS

KM and KN wrote the manuscript.

## Conflict of Interest Statement

The authors declare that the research was conducted in the absence of any commercial or financial relationships that could be construed as a potential conflict of interest.

## References

[B1] Albertsson A. M. Zhang X. Vontell R. Bi D. Bronson R. T. Supramaniam V. (2018). gammadelta T cells contribute to injury in the developing brain. *Am. J. Pathol.* 188 757–767. 10.1016/j.ajpath.2017.11.012 29248460 PMC5840494

[B2] Arango Duque G. Descoteaux A. (2014). Macrophage cytokines: involvement in immunity and infectious diseases. *Front. Immunol.* 5:491. 10.3389/fimmu.2014.00491 25339958 PMC4188125

[B3] Barco A. Patterson S. L. Alarcon J. M. Gromova P. Mata-Roig M. Morozov A. (2005). Gene expression profiling of facilitated L-LTP in VP16-CREB mice reveals that BDNF is critical for the maintenance of LTP and its synaptic capture. *Neuron* 48 123–137. 10.1016/j.neuron.2005.09.005 16202713

[B4] Baudouin S. J. Angibaud J. Loussouarn G. Bonnamain V. Matsuura A. Kinebuchi M. (2008). The signaling adaptor protein CD3zeta is a negative regulator of dendrite development in young neurons. *Mol. Biol. Cell* 19 2444–2456. 10.1091/mbc.E07-09-0947 18367546 PMC2397320

[B5] Bauer S. Kerr B. J. Patterson P. H. (2007). The neuropoietic cytokine family in development, plasticity, disease and injury. *Nat. Rev. Neurosci.* 8 221–232. 10.1038/nrn2054 17311007

[B6] Baumgarth N. (2011). The double life of a B-1 cell: self-reactivity selects for protective effector functions. *Nat. Rev. Immunol.* 11 34–46. 10.1038/nri2901 21151033

[B7] Bjartmar L. Huberman A. D. Ullian E. M. Renteria R. C. Liu X. Xu W. (2006). Neuronal pentraxins mediate synaptic refinement in the developing visual system. *J. Neurosci.* 26 6269–6281. 10.1523/JNEUROSCI.4212-05.2006 16763034 PMC2579897

[B8] Blum J. S. Wearsch P. A. Cresswell P. (2013). Pathways of antigen processing. *Annu. Rev. Immunol.* 31 443–473. 10.1146/annurev-immunol-032712-095910 23298205 PMC4026165

[B9] Boulanger L. M. (2009). Immune proteins in brain development and synaptic plasticity. *Neuron* 64 93–109. 10.1016/j.neuron.2009.09.001 19840552

[B10] Bruhns P. (2012). Properties of mouse and human IgG receptors and their contribution to disease models. *Blood* 119 5640–5649. 10.1182/blood-2012-01-380121 22535666

[B11] Bryceson Y. T. Foster J. A. Kuppusamy S. P. Herkenham M. Long E. O. (2005). Expression of a killer cell receptor-like gene in plastic regions of the central nervous system. *J. Neuroimmunol.* 161 177–182. 10.1016/j.jneuroim.2004.11.018 15748957

[B12] Bulloch K. Miller M. M. Gal-Toth J. Milner T. A. Gottfried-Blackmore A. Waters E. M. (2008). CD11c/EYFP transgene illuminates a discrete network of dendritic cells within the embryonic, neonatal, adult, and injured mouse brain. *J. Comp. Neurol.* 508 687–710. 10.1002/cne.21668 18386786

[B13] Call M. E. Wucherpfennig K. W. (2007). Common themes in the assembly and architecture of activating immune receptors. *Nat. Rev. Immunol.* 7 841–850. 10.1038/nri2186 17960150

[B14] Choi G. B. Yim Y. S. Wong H. Kim S. Kim H. Kim S. V. (2016). The maternal interleukin-17a pathway in mice promotes autism-like phenotypes in offspring. *Science* 351 933–939. 10.1126/science.aad0314 26822608 PMC4782964

[B15] Cunningham C. L. Martinez-Cerdeno V. Noctor S. C. (2013). Microglia regulate the number of neural precursor cells in the developing cerebral cortex. *J. Neurosci.* 33 4216–4233. 10.1523/JNEUROSCI.3441-12.2013 23467340 PMC3711552

[B16] Da Mesquita S. Louveau A. Vaccari A. Smirnov I. Cornelison R. C. Kingsmore K. M. (2018). Functional aspects of meningeal lymphatics in ageing and Alzheimer’s disease. *Nature* 560 185–191. 10.1038/s41586-018-0368-8 30046111 PMC6085146

[B17] Datwani A. Mcconnell M. J. Kanold P. O. Micheva K. D. Busse B. Shamloo M. (2009). Classical MHCI molecules regulate retinogeniculate refinement and limit ocular dominance plasticity. *Neuron* 64 463–470. 10.1016/j.neuron.2009.10.015 19945389 PMC2787480

[B18] De S. Van Deren D. Peden E. Hockin M. Boulet A. Titen S. (2018). Two distinct ontogenies confer heterogeneity to mouse brain microglia. *Development* 145:dev152306. 10.1242/dev.152306 29973370 PMC6053660

[B19] Derecki N. C. Cardani A. N. Yang C. H. Quinnies K. M. Crihfield A. Lynch K. R. (2010). Regulation of learning and memory by meningeal immunity: a key role for IL-4. *J. Exp. Med.* 207 1067–1080. 10.1084/jem.20091419 20439540 PMC2867291

[B20] Elmer B. M. McAllister A. K. (2012). Major histocompatibility complex class I proteins in brain development and plasticity. *Trends Neurosci.* 35 660–670. 10.1016/j.tins.2012.08.001 22939644 PMC3493469

[B21] Estes M. L. McAllister A. K. (2016). Maternal immune activation: implications for neuropsychiatric disorders. *Science* 353 772–777. 10.1126/science.aag3194 27540164 PMC5650490

[B22] Fantin A. Vieira J. M. Gestri G. Denti L. Schwarz Q. Prykhozhij S. (2010). Tissue macrophages act as cellular chaperones for vascular anastomosis downstream of VEGF-mediated endothelial tip cell induction. *Blood* 116 829–840. 10.1182/blood-2009-12-257832 20404134 PMC2938310

[B23] Fossati G. Pozzi D. Canzi A. Mirabella F. Valentino S. Morini R. (2019). Pentraxin 3 regulates synaptic function by inducing AMPA receptor clustering via ECM remodeling and beta1-integrin. *EMBO J.* 38:e99529. 10.15252/embj.201899529 30396995 PMC6315291

[B24] Garlanda C. Bottazzi B. Bastone A. Mantovani A. (2005). Pentraxins at the crossroads between innate immunity, inflammation, matrix deposition, and female fertility. *Annu. Rev. Immunol.* 23 337–366. 10.1146/annurev.immunol.23.021704.115756 15771574

[B25] Ginhoux F. Greter M. Leboeuf M. Nandi S. See P. Gokhan S. (2010). Fate mapping analysis reveals that adult microglia derive from primitive macrophages. *Science* 330 841–845. 10.1126/science.1194637 20966214 PMC3719181

[B26] Guyon A. (2014). CXCL12 chemokine and its receptors as major players in the interactions between immune and nervous systems. *Front. Cell. Neurosci.* 8:65. 10.3389/fncel.2014.00065 24639628 PMC3944789

[B27] Huh G. S. Boulanger L. M. Du H. Riquelme P. A. Brotz T. M. Shatz C. J. (2000). Functional requirement for class I MHC in CNS development and plasticity. *Science* 290 2155–2159. 10.1126/science.290.5499.2155 11118151 PMC2175035

[B28] Imai T. Hieshima K. Haskell C. Baba M. Nagira M. Nishimura M. (1997). Identification and molecular characterization of fractalkine receptor CX3CR1, which mediates both leukocyte migration and adhesion. *Cell* 91 521–530. 10.1016/S0092-8674(00)80438-9 9390561

[B29] Imai T. Yamazaki T. Kobayakawa R. Kobayakawa K. Abe T. Suzuki M. (2009). Pre-target axon sorting establishes the neural map topography. *Science* 325 585–590. 10.1126/science.1173596 19589963

[B30] Jeon H. Lee S. Lee W. H. Suk K. (2010). Analysis of glial secretome: the long pentraxin PTX3 modulates phagocytic activity of microglia. *J. Neuroimmunol.* 229 63–72. 10.1016/j.jneuroim.2010.07.001 20674043

[B31] Kawai T. Akira S. (2007). TLR signaling. *Semin. Immunol.* 19 24–32. 10.1016/j.smim.2006.12.004 17275323

[B32] Kettenmann H. Kirchhoff F. Verkhratsky A. (2013). Microglia: new roles for the synaptic stripper. *Neuron* 77 10–18. 10.1016/j.neuron.2012.12.023 23312512

[B33] Khariv V. Pang K. Servatius R. J. David B. T. Goodus M. T. Beck K. D. (2013). Toll-like receptor 9 deficiency impacts sensory and motor behaviors. *Brain Behav. Immun.* 32 164–172. 10.1016/j.bbi.2013.04.007 23624295

[B34] Kim S. Kim H. Yim Y. S. Ha S. Atarashi K. Tan T. G. (2017). Maternal gut bacteria promote neurodevelopmental abnormalities in mouse offspring. *Nature* 549 528–532. 10.1038/nature23910 28902840 PMC5870873

[B35] Kim S. Y. Yasuda S. Tanaka H. Yamagata K. Kim H. (2011). Non-clustered protocadherin. *Cell Adh. Migr.* 5 97–105. 10.4161/cam.5.2.14374 21173574 PMC3084973

[B36] Kioussis D. Pachnis V. (2009). Immune and nervous systems: more than just a superficial similarity? *Immunity* 31 705–710. 10.1016/j.immuni.2009.09.009 19836266

[B37] Kipnis J. Cohen H. Cardon M. Ziv Y. Schwartz M. (2004). T cell deficiency leads to cognitive dysfunction: implications for therapeutic vaccination for schizophrenia and other psychiatric conditions. *Proc. Natl. Acad. Sci. U.S.A.* 101 8180–8185. 10.1073/pnas.0402268101 15141078 PMC419577

[B38] Klein R. (2009). Bidirectional modulation of synaptic functions by Eph/ephrin signaling. *Nat. Neurosci.* 12 15–20. 10.1038/nn.2231 19029886

[B39] Knuesel I. Chicha L. Britschgi M. Schobel S. A. Bodmer M. Hellings J. A. (2014). Maternal immune activation and abnormal brain development across CNS disorders. *Nat. Rev. Neurol.* 10 643–660. 10.1038/nrneurol.2014.187 25311587

[B40] Korin B. Ben-Shaanan T. L. Schiller M. Dubovik T. Azulay-Debby H. Boshnak N. T. (2017). High-dimensional, single-cell characterization of the brain’s immune compartment. *Nat. Neurosci.* 20 1300–1309. 10.1038/nn.4610 28758994

[B41] Kuhns M. S. Davis M. M. Garcia K. C. (2006). Deconstructing the form and function of the TCR/CD3 complex. *Immunity* 24 133–139. 10.1016/j.immuni.2006.01.006 16473826

[B42] Kumanogoh A. Kikutani H. (2013). Immunological functions of the neuropilins and plexins as receptors for semaphorins. *Nat. Rev. Immunol.* 13 802–814. 10.1038/nri3545 24319778

[B43] Laidlaw B. J. Schmidt T. H. Green J. A. Allen C. D. Okada T. Cyster J. G. (2017). The Eph-related tyrosine kinase ligand Ephrin-B1 marks germinal center and memory precursor B cells. *J. Exp. Med.* 214 639–649. 10.1084/jem.20161461 28143955 PMC5339677

[B44] Lee H. Brott B. K. Kirkby L. A. Adelson J. D. Cheng S. Feller M. B. (2014). Synapse elimination and learning rules co-regulated by MHC class I H2-Db. *Nature* 509 195–200. 10.1038/nature13154 24695230 PMC4016165

[B45] Li M. Ransohoff R. M. (2008). Multiple roles of chemokine CXCL12 in the central nervous system: a migration from immunology to neurobiology. *Prog. Neurobiol.* 84 116–131. 10.1016/j.pneurobio.2007.11.003 18177992 PMC2324067

[B46] Louveau A. Smirnov I. Keyes T. J. Eccles J. D. Rouhani S. J. Peske J. D. (2015). Structural and functional features of central nervous system lymphatic vessels. *Nature* 523 337–341. 10.1038/nature14432 26030524 PMC4506234

[B47] Lu P. Shih C. Qi H. (2017). Ephrin B1-mediated repulsion and signaling control germinal center T cell territoriality and function. *Science* 356:eaai9264. 10.1126/science.aai9264 28408722

[B48] Luo C. Keown C. L. Kurihara L. Zhou J. He Y. Li J. (2017). Single-cell methylomes identify neuronal subtypes and regulatory elements in mammalian cortex. *Science* 357 600–604. 10.1126/science.aan3351 28798132 PMC5570439

[B49] McAllister A. K. (2014). Major histocompatibility complex I in brain development and schizophrenia. *Biol. Psychiatry* 75 262–268. 10.1016/j.biopsych.2013.10.003 24199663 PMC4354937

[B50] Miyamoto A. Wake H. Ishikawa A. W. Eto K. Shibata K. Murakoshi H. (2016). Microglia contact induces synapse formation in developing somatosensory cortex. *Nat. Commun.* 7:12540. 10.1038/ncomms12540 27558646 PMC5007295

[B51] Nagasawa T. (2015). CXCL12/SDF-1 and CXCR4. *Front. Immunol.* 6:301. 10.3389/fimmu.2015.00301 26124757 PMC4464259

[B52] Nakahara J. Seiwa C. Shibuya A. Aiso S. Asou H. (2003a). Expression of Fc receptor for immunoglobulin M in oligodendrocytes and myelin of mouse central nervous system. *Neurosci. Lett.* 337 73–76. 10.1016/s0304-3940(02)01312-5 12527391

[B53] Nakahara J. Tan-Takeuchi K. Seiwa C. Gotoh M. Kaifu T. Ujike A. (2003b). Signaling via immunoglobulin Fc receptors induces oligodendrocyte precursor cell differentiation. *Dev. Cell* 4 841–852. 10.1016/S1534-5807(03)00155-2 12791269

[B54] Nakamura K. Hirai H. Torashima T. Miyazaki T. Tsurui H. Xiu Y. (2007). CD3 and immunoglobulin G Fc receptor regulate cerebellar functions. *Mol. Cell Biol.* 27 5128–5134. 10.1128/MCB.01072-06 17502348 PMC1951947

[B55] Nazmi A. Albertsson A. M. Rocha-Ferreira E. Zhang X. Vontell R. Zelco A. (2018). Lymphocytes contribute to the pathophysiology of neonatal brain injury. *Front. Neurol.* 9:159. 10.3389/fneur.2018.00159 29615958 PMC5868390

[B56] Nishide M. Kumanogoh A. (2018). The role of semaphorins in immune responses and autoimmune rheumatic diseases. *Nat. Rev. Rheumatol.* 14 19–31. 10.1038/nrrheum.2017.201 29213125

[B57] Oberheim N. A. Wang X. Goldman S. Nedergaard M. (2006). Astrocytic complexity distinguishes the human brain. *Trends Neurosci.* 29 547–553. 10.1016/j.tins.2006.08.004 16938356

[B58] Okun E. Griffioen K. J. Mattson M. P. (2011). Toll-like receptor signaling in neural plasticity and disease. *Trends Neurosci.* 34 269–281. 10.1016/j.tins.2011.02.005 21419501 PMC3095763

[B59] Ott J. A. Castro C. D. Deiss T. C. Ohta Y. Flajnik M. F. Criscitiello M. F. (2018). Somatic hypermutation of T cell receptor alpha chain contributes to selection in nurse shark thymus. *eLife* 7:e28477. 10.7554/eLife.28477 29664399 PMC5931798

[B60] Pang Y. Fan L. W. Tien L. T. Dai X. Zheng B. Cai Z. (2013). Differential roles of astrocyte and microglia in supporting oligodendrocyte development and myelination in vitro. *Brain Behav.* 3 503–514. 10.1002/brb3.152 24392271 PMC3869978

[B61] Paolicelli R. C. Bisht K. Trembley M. -È (2014). Fractalkine regulation of microglial physiology and consequences on the brain and behavior. *Front. Cell. Neurosci.* 8:129. 10.3389/fncel.2014.00129 24860431 PMC4026677

[B62] Pelkey K. A. Barksdale E. Craig M. T. Yuan X. Sukumaran M. Vargish G. A. (2015). Pentraxins coordinate excitatory synapse maturation and circuit integration of parvalbumin interneurons. *Neuron* 85 1257–1272. 10.1016/j.neuron.2015.02.020 25754824 PMC4368480

[B63] Pont-Lezica L. Beumer W. Colasse S. Drexhage H. Versnel M. Bessis A. (2014). Microglia shape corpus callosum axon tract fasciculation: functional impact of prenatal inflammation. *Eur. J. Neurosci.* 39 1551–1557. 10.1111/ejn.12508 24593277

[B64] Pösel C. Möller K. Boltze J. Wagner D. C. Weise G. (2016). Isolation and flow cytometric analysis of immune cells from the ischemic mouse brain. *J. Vis. Exp.* 108:53658. 10.3791/53658 26967380 PMC4828148

[B65] Radjavi A. Smirnov I. Kipnis J. (2014). Brain antigen-reactive CD4+ T cells are sufficient to support learning behavior in mice with limited T cell repertoire. *Brain Behav. Immun.* 35 58–63. 10.1016/j.bbi.2013.08.013 24012647 PMC3858511

[B66] Ransohoff R. M. (2009). Chemokines and chemokine receptors: standing at the crossroads of immunobiology and neurobiology. *Immunity* 31 711–721. 10.1016/j.immuni.2009.09.010 19836265 PMC2787682

[B67] Reemst K. Noctor S. C. Lucassen P. J. Hol E. M. (2016). The indispensable roles of microglia and astrocytes during brain development. *Front. Hum. Neurosci.* 10:566. 10.3389/fnhum.2016.00566 27877121 PMC5099170

[B68] Ricklin D. Hajishengallis G. Yang K. Lambris J. D. (2010). Complement: a key system for immune surveillance and homeostasis. *Nat. Immunol.* 11 785–797. 10.1038/ni.1923 20720586 PMC2924908

[B69] Rilett K. C. Friedel M. Ellegood J. Mackenzie R. N. Lerch J. P. Foster J. A. (2015). Loss of T cells influences sex differences in behavior and brain structure. *Brain Behav. Immun.* 46 249–260. 10.1016/j.bbi.2015.02.016 25725160

[B70] Rosser E. C. Mauri C. (2015). Regulatory B cells: origin, phenotype, and function. *Immunity* 42 607–612. 10.1016/j.immuni.2015.04.005 25902480

[B71] Rudolph M. D. Graham A. M. Feczko E. Miranda-Dominguez O. Rasmussen J. M. Nardos R. (2018). Maternal IL-6 during pregnancy can be estimated from newborn brain connectivity and predicts future working memory in offspring. *Nat. Neurosci.* 21 765–772. 10.1038/s41593-018-0128-y 29632361 PMC5920734

[B72] Sakaguchi S. Yamaguchi T. Nomura T. Ono M. (2008). Regulatory T cells and immune tolerance. *Cell* 133 775–787. 10.1016/j.cell.2008.05.009 18510923

[B73] Schmucker D. Chen B. (2009). Dscam and DSCAM: complex genes in simple animals, complex animals yet simple genes. *Genes Dev.* 23 147–156. 10.1101/gad.1752909 19171779

[B74] Shibuya A. Sakamoto N. Shimizu Y. Shibuya K. Osawa M. Hiroyama T. (2000). Fc alpha/mu receptor mediates endocytosis of IgM-coated microbes. *Nat. Immunol.* 1 441–446. 10.1038/80886 11062505

[B75] Shigemoto-Mogami Y. Hoshikawa K. Goldman J. E. Sekino Y. Sato K. (2014). Microglia enhance neurogenesis and oligodendrogenesis in the early postnatal subventricular zone. *J. Neurosci.* 34 2231–2243. 10.1523/JNEUROSCI.1619-13.2014 24501362 PMC3913870

[B76] Shin Yim Y. Park A. Berrios J. Lafourcade M. Pascual L. M. Soares N. (2017). Reversing behavioural abnormalities in mice exposed to maternal inflammation. *Nature* 549 482–487. 10.1038/nature23909 28902835 PMC5796433

[B77] Smith S. E. Li J. Garbett K. Mirnics K. Patterson P. H. (2007). Maternal immune activation alters fetal brain development through interleukin-6. *J. Neurosci.* 27 10695–10702. 10.1523/JNEUROSCI.2178-07.2007 17913903 PMC2387067

[B78] Song C. Nicholson J. D. Clark S. M. Li X. Keegan A. D. Tonelli L. H. (2016). Expansion of brain T cells in homeostatic conditions in lymphopenic Rag2(-/-) mice. *Brain Behav. Immun.* 57 161–172. 10.1016/j.bbi.2016.03.017 27013354 PMC5010944

[B79] Southwell D. G. Paredes M. F. Galvao R. P. Jones D. L. Froemke R. C. Sebe J. Y. (2012). Intrinsically determined cell death of developing cortical interneurons. *Nature* 491 109–113. 10.1038/nature11523 23041929 PMC3726009

[B80] Squarzoni P. Oller G. Hoeffel G. Pont-Lezica L. Rostaing P. Low D. (2014). Microglia modulate wiring of the embryonic forebrain. *Cell Rep.* 8 1271–1279. 10.1016/j.celrep.2014.07.042 25159150

[B81] Stephan A. H. Barres B. A. Stevens B. (2012). The complement system: an unexpected role in synaptic pruning during development and disease. *Annu. Rev. Neurosci.* 35 369–389. 10.1146/annurev-neuro-061010-113810 22715882

[B82] Stevens B. Allen N. J. Vazquez L. E. Howell G. R. Christopherson K. S. Nouri N. (2007). The classical complement cascade mediates CNS synapse elimination. *Cell* 131 1164–1178. 10.1016/j.cell.2007.10.036 18083105

[B83] Südhof T. C. (2017). Synaptic neurexin complexes: a molecular code for the logic of neural circuits. *Cell* 171 745–769. 10.1016/j.cell.2017.10.024 29100073 PMC5694349

[B84] Syken J. Grandpre T. Kanold P. O. Shatz C. J. (2006). PirB restricts ocular-dominance plasticity in visual cortex. *Science* 313 1795–1800. 10.1126/science.1128232 16917027

[B85] Syken J. Shatz C. J. (2003). Expression of T cell receptor beta locus in central nervous system neurons. *Proc. Natl. Acad. Sci. U.S.A.* 100 13048–13053. 10.1073/pnas.1735415100 14569018 PMC240742

[B86] Takai T. (2005). Paired immunoglobulin-like receptors and their MHC class I recognition. *Immunology* 115 433–440. 10.1111/j.1365-2567.2005.02177.x 16011512 PMC1782189

[B87] Tanabe S. Yamashita T. (2018a). B lymphocytes: crucial contributors to brain development and neurological diseases. *Neurosci. Res.* 139 37–41. 10.1016/j.neures.2018.07.002 30009855

[B88] Tanabe S. Yamashita T. (2018b). B-1a lymphocytes promote oligodendrogenesis during brain development. *Nat. Neurosci.* 21 506–516. 10.1038/s41593-018-0106-4 29507409

[B89] Tran T. S. Kolodkin A. L. Bharadwaj R. (2007). Semaphorin regulation of cellular morphology. *Annu. Rev. Cell Dev. Biol.* 23 263–292. 10.1146/annurev.cellbio.22.010605.093554 17539753

[B90] Ueno M. Fujita Y. Tanaka T. Nakamura Y. Kikuta J. Ishii M. (2013). Layer V cortical neurons require microglial support for survival during postnatal development. *Nat. Neurosci.* 16 543–551. 10.1038/nn.3358 23525041

[B91] Upender M. B. Dunn J. A. Wilson S. M. Naegela J. R. (1997). Immunoglobulin molecules are present in early-generated neuronal populations in the rat cerebral cortex and retina. *J. Comp. Neurol.* 384 271–282. 10.1002/(sici)1096-9861(19970728)384:2<271::aid-cne7>3.0.co;2-z 9215722

[B92] Vainchtein I. D. Chin G. Cho F. S. Kelley K. W. Miller J. G. Chien E. C. (2018). Astrocyte-derived interleukin-33 promotes microglial synapse engulfment and neural circuit development. *Science* 359 1269–1273. 10.1126/science.aal3589 29420261 PMC6070131

[B93] Vazquez-Cintron E. J. Monu N. R. Burns J. C. Blum R. Chen G. Lopez P. (2012). Protocadherin-18 is a novel differentiation marker and an inhibitory signaling receptor for CD8+ effector memory T cells. *PLoS One* 7:e36101. 10.1371/journal.pone.0036101 22567129 PMC3342238

[B94] Veerhuis R. Nielsen H. M. Tenner A. J. (2011). Complement in the brain. *Mol. Immunol.* 48 1592–1603. 10.1016/j.molimm.2011.04.003 21546088 PMC3142281

[B95] Vivier E. Raulet D. H. Moretta A. Caligiuri M. A. Zitvogel L. Lanier L. I. (2011). Innate or Adaptive Immunity? The Example of Natural Killer Cells. *Science* 331 44–49. 10.1126/science.1198687 21212348 PMC3089969

[B96] Watson F. L. Püttmann-Holgado R. Thomas F. Lamar D. L. Hughes M. Kondo M. (2005). Extensive diversity of Ig-superfamily proteins in the immune system of insects. *Science* 309 1874–1878. 10.1126/science.1116887 16109846

[B97] Williams J. L. Holman D. W. Klein R. S. (2014). Chemokines in the balance: maintenance of homeostasis and protection at CNS barriers. *Front. Cell. Neurosci.* 8:154. 10.3389/fncel.2014.00154 24920943 PMC4036130

[B98] Wolf S. A. Steiner B. Akpinarli A. Kammertoens T. Nassenstein C. Braun A. (2009). CD4-positive T lymphocytes provide a neuroimmunological link in the control of adult hippocampal neurogenesis. *J. Immunol.* 182 3979–3984. 10.4049/jimmunol.0801218 19299695

[B99] Wong F. K. Bercsenyi K. Sreenivasan V. Portales A. Fernandez-Otero M. Marin O. (2018). Pyramidal cell regulation of interneuron survival sculpts cortical networks. *Nature* 557 668–673. 10.1038/s41586-018-0139-6 29849154 PMC6207348

[B100] Wu W. L. Hsiao E. Y. Yan Z. Mazmanian S. K. Patterson P. H. (2017). The placental interleukin-6 signaling controls fetal brain development and behavior. *Brain Behav. Immun.* 62 11–23. 10.1016/j.bbi.2016.11.007 27838335 PMC5373986

[B101] Wu Y. Dissing-Olesen L. Macvicar B. A. Stevens B. (2015). Microglia: dynamic mediators of synapse development and plasticity. *Trends Immunol.* 36 605–613. 10.1016/j.it.2015.08.008 26431938 PMC4841266

[B102] Xu H. P. Chen H. Ding Q. Xie Z. H. Chen L. Diao L. (2010). The immune protein CD3zeta is required for normal development of neural circuits in the retina. *Neuron* 65 503–515. 10.1016/j.neuron.2010.01.035 20188655 PMC3037728

[B103] Yagi T. (2008). Clustered protocadherin family. *Dev. Growth Differ.* 50 S131–S140. 10.1111/j.1440-169X.2008.00991.x 18430161

[B104] Yang D. Sun Y. Y. Bhaumik S. K. Li Y. Baumann J. M. Lin X. (2014). Blocking lymphocyte trafficking with FTY720 prevents inflammation-sensitized hypoxic-ischemic brain injury in newborns. *J. Neurosci.* 34 16467–16481. 10.1523/JNEUROSCI.2582-14.2014 25471584 PMC4252554

[B105] Yizhar O. Fenno L. E. Prigge M. Schneider F. Davidson T. J. O’shea D. J. (2011). Neocortical excitation/inhibition balance in information processing and social dysfunction. *Nature* 477 171–178. 10.1038/nature10360 21796121 PMC4155501

[B106] Yuzaki M. (2017). The C1q complement family of synaptic organizers: not just complementary. *Curr. Opin. Neurobiol.* 45 9–15. 10.1016/j.conb.2017.02.002 28219683

[B107] Zalc B. Goujet D. Colman D. (2008). The origin of the myelination program in vertebrates. *Curr. Biol.* 18 R511–R512. 10.1016/j.cub.2008.04.010 18579089

[B108] Zhang X. Rocha-Ferreira E. Li T. Vontell R. Jabin D. Hua S. (2017). gammadeltaT cells but not alphabetaT cells contribute to sepsis-induced white matter injury and motor abnormalities in mice. *J. Neuroinflammation* 14:255. 10.1186/s12974-017-1029-9 29262837 PMC5738716

[B109] Zhu Y. Murakami F. (2012). Chemokine CXCL12 and its receptors in the developing central nervous system: emerging themes and future perspectives. *Dev. Neurobiol.* 72 1349–1362. 10.1002/dneu.22041 22689506

[B110] Ziv Y. Ron N. Butovsky O. Landa G. Sudai E. Greenberg N. (2006). Immune cells contribute to the maintenance of neurogenesis and spatial learning abilities in adulthood. *Nat. Neurosci.* 9 268–275. 10.1038/nn1629 16415867

[B111] Zohar O. Reiter Y. Bennink J. R. Lev A. Cavallaro S. Paratore S. (2008). Cutting edge: MHC class I-Ly49 interaction regulates neuronal function. *J. Immunol.* 180 6447–6451. 10.4049/jimmunol.180.10.6447 18453559 PMC2727684

